# DMBA-Induced Oral Carcinoma in Syrian Hamster: Increased Carcinogenic Effect by Dexamethasone Coexposition

**DOI:** 10.1155/2020/1470868

**Published:** 2020-02-13

**Authors:** Diana A. Martínez B., Paola Andrea Barato Gómez, Carlos Arturo Iregui Castro, Jaiver E. Rosas Pérez

**Affiliations:** ^1^Department of Pharmacy, Sciences Faculty, Universidad Nacional de Colombia, Bogotá 111321, Colombia; ^2^Corporación Patología Veterinaria (CORPAVET), Bogotá 111321, Colombia; ^3^Group of Veterinary Pathobiology, Universidad Nacional de Colombia, Bogotá 111321, Colombia

## Abstract

**Objectives:**

To investigate the effect of systemic administration of the immunosuppressant dexamethasone (DM) while inducing hamster buccal pouch DMBA carcinogenesis. *Materials and Methods*. Two different experiments were performed. In the first experiment, hamsters' right buccal pouches in group A (*n* = 10) were painted three times per week with 7,12-dimethylbenzanthracene (DMBA) 0.5%, while pouches of animals in group B (*n* = 10) were painted three times per week with 7,12-dimethylbenzanthracene (DMBA) 0.5%, while pouches of animals in group B (

**Results:**

The time of macroscopic neoplasm development was reduced when DM-DMBA coexposition was employed, finding tumors after 10–12 weeks of exposition. In addition, the frequency of histopathological lesions was higher.

**Conclusion:**

Immunomodulatory action of dexamethasone may reduce the time of oral squamous cell carcinoma (OSCC) induction and may increase the incidence of neoplasms developed.

## 1. Introduction

Animal models are important to identify the etiology of different human diseases under controlled environment and rigorous supervision [1]. Furthermore, the use of animal models is part of the preclinical phases while testing new drugs before their trial on human beings [2]. Specifically, in the case of the oral squamous cell carcinoma (OSCC) study, animal models have been important to identify the stages of the malignancy prior to a carcinoma generation [3]. To understand the factors involved in the process of carcinogenesis, the ideal animal model would undergo a spontaneous cancerization as it occurs in human beings, but natural oral carcinoma cases are too low in laboratory animals [4]. Different animal models of OSCC have been proposed to study risk factors, to identify biomarkers, and to test preventive and curative treatments [3, 5, 6].

Hamster buccal pouch carcinogenesis induced by painting with different carcinogenic agents in the oral mucosa has been widely studied for determination of histochemical, genetic, and biomolecular changes through the disease [7–9]. Given the similarity of the carcinogenesis process between humans and hamsters, this animal model is an important tool for research in oral oncology [10]. Salley in 1954 was the first to propose the experimental conditions to successfully develop the OSCC in hamsters [11]; after that, a variety of protocols have been described with the same purpose. Differences in the age of the hamsters at the beginning of tumor induction, different solvents used in the carcinogenic solution, and different times to tumor development are some of the variables that may change between models [12]. Following any of the protocols described, morphopathological changes such as hyperkeratosis, hyperplasia, dysplasia, invasion, and differentiated OSCC have been identified through the carcinogenesis process [13]. During the first years of study of this carcinogenesis model, the immunological status of the animal was compromised to develop more invasive and metastatic carcinomas [14]. For example, the anti-hamster lymphocyte serum was injected during the application of DMBA to the hamster buccal pouch to increase the tumor burden and to generate deeper invasion and more anaplastic cells than the control groups [15]. Also, cortisone was used as an immunomodulatory agent to inhibit the leukocyte response to the carcinogenic agent DMBA, and animals that were injected with 2.0 mg of cortisone acetate three times weekly developed an oral carcinoma more invasively and with larger tumors [16, 17]. Corticoids were used as well to develop metastasis to cervical nodes and further organs such as lungs [18]. However, actually it is on debate whether immunosuppressive drugs influence neoplasia development. Some of the concluding results indicate that systemic administration of corticoids may enhance the progress of squamous cell carcinoma (including skin carcinoma) [19]. Although a unique protocol has not been established for DMBA-induced carcinogenesis including corticoids or immunomodulatory agents, this complementary treatment could increase the carcinogenic effect of chemical exposition, particularly when genetically modified rodents are not available.

Herein, the present study is intended to evaluate the difference between two experiments conducted at different times, involving the systemic administration of the corticosteroid dexamethasone as an enhancer in the DMBA-induced carcinogenesis model on the hamster buccal pouch. At the end of the animal model implementation, by running a third experiment, it was possible to successfully generate macroscopic tumors on hamster cheek pouches of 15 out of 18 animals exposed to DMBA local application and concomitant subcutaneous DM administration.

## 2. Materials and Methods

### 2.1. Animal Housing

Four-week-old outbred male Syrian hamsters (*Mesocricetus auratus*) were procured from Instituto Nacional de Salud, Bogotá, Colombia. The hamsters were housed in filter-capped polypropylene cages (RAIR OneCage® system) at controlled temperature (23 ± 2°C) and maintained on a 12 h light/dark cycle. Water and autoclavable diet pellets (LabDiet 5010®) were given ad libitum. Before beginning each experiment, animals were housed for 2 weeks in a quarantine time; at that time, hematological and parasitological tests were performed in order to evaluate the health status of the animals. If experimentation sedation was needed, 1 mg/kg acepromazine was peritoneally injected. All animal procedures were made conforming to “Guide for the Care and Use of Laboratory Animals” [20] and national laws [21] and were approved by the Ethics Committee of the Sciences Faculty of Universidad Nacional de Colombia.

### 2.2. Carcinogenesis Induction

Two different experiments were performed at different times. After quarantine time, in the first experiment, a total number of 14 animals (six weeks old) were divided into 2 groups. Group A (*n* = 10) was exposed by painting the right buccal pouch three times per week with a camel-hair brush soaked in 0.5% 7,12-dimethylbenzanthracene (DMBA, Sigma®) dissolved in mineral oil. Group B (*n* = 4) was painted with only mineral oil, and no DMBA, for the same three times per week. In the second experiment, the same number of animals and groups was used, but subcutaneous dexamethasone phosphate solution of dose 1 mg/kg BW (high dose of corticoid bearing in mind the concentration needed for the immunosuppressive effect [22]) was injected in all 14 animals (including DMBA- and no DMBA-exposed hamsters) for seven days every three weeks. Macroscopically, tumors were measured using a digital caliper, and their volume was calculated using the formula *V* = 0.5*∗*(length)(width)^2^ [23]. Presence of gross lesions was evaluated, which included mucosal thickness, exudation, ulcers, and tumors. All lesions were evaluated under the supervision of a qualified veterinary pathologist. Additionally, weight of animals was recorded every week. Hamsters were euthanized if the tumor reached a maximum volume (200 mm^3^) or if there was a weight loss over 20% in a week.

### 2.3. Histopathological Evaluation

After three weeks of DMBA exposition, 2 animals were sacrificed by an intraperitoneal overdose of pentobarbital and diphenylhydantoin (Euthanex®), and samples from tongue, esophagus, and vital organs (brain, heart, lungs, stomach, liver, spleen, kidneys, adrenal glands, and intestines) were taken for histopathological evaluation. Sacrifices of 2 animals at a time were performed at weeks 3, 6, 9, 12, and 15. These times were selected randomly in order to evaluate the carcinogenesis progress. Animals from the control group (group B) were sacrificed by pairs at weeks 9 and 15. All samples were fixed in buffered formalin 3.7%, processed for routine H&E staining, and microscopically evaluated (standard optical microscope Olympus® CX21). A categorical nomination was determined when these pathological lesions were present: hyperkeratotic cell presence, ulceration, exocytosis, necrosis, dysplasia, metaplasia, papilloma, carcinoma, and infiltration of lymphocytic cells. Analysis was performed by one blinding veterinary pathologist.

## 3. Results

### 3.1. Clinical Evaluation

**Figure 1:**
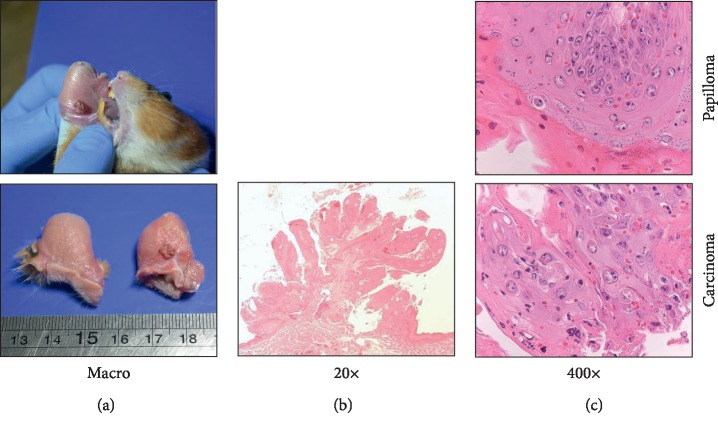
Tumor found on the hamster pouch after 14 weeks of DMBA exposition and subsequent s.c. administration of DM 1 mg/kg for seven days. Volume: 14.3 mm^3^.

During first two weeks of adaptation, hematological and parasitic analyses were performed; no pathological disorders or infections were found in any of the animals used. In the first experiment with no injection of dexamethasone, the mucosa of the DMBA-exposed pouch (100% of exposed animals) became red velveted with a rough appearance (thickening of the mucosa), compared to the control group's pouch after four weeks of exposition. After 12 weeks of DMBA exposition and the sacrifice of 8 animals with no macroscopic evidence of ulceration, papilloma, necrosis, or tumors, the last two animals were s.c. injected with dexamethasone phosphate solution (dose 1 mg/kg). After two weeks of immunosuppression (a total of 14 weeks of DMBA exposition), one of the hamsters developed an exophytic tumor of 14.3 mm^3^ volume with a red rough appearance (Figure 1). Due to changes in the behavior of the animal, vocalization during the manipulation and mucosa bleeding, this hamster was euthanized and samples for histological evaluation were taken. The last animal in experimentation was exposed to DMBA up to 17 weeks with no relevant macroscopic findings. At the time of necropsy, a red velveted erythematous lesion was found on the inner side of the exposed pouch with a diameter of 2 mm approx. Body weight of animals did not show any evidence of inanition or disease state, and only the animal which developed the exophytic tumor had diminished body weight by 11% in a week, evidencing illness and an interference of the neoplastic lesion with its food intake. All control animals were healthy during the experiment.

**Figure 2:**
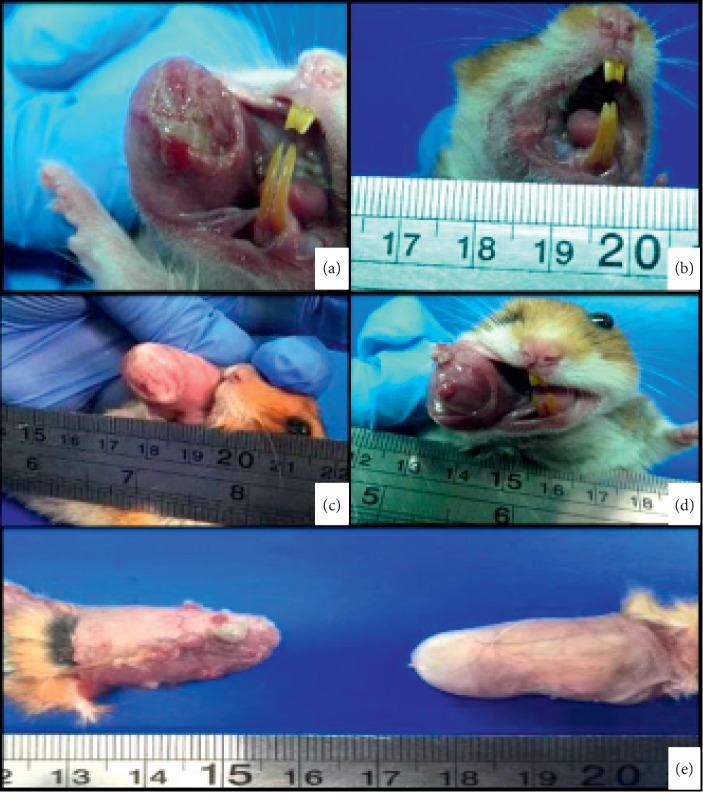
Hamsters from the second experiment exposed to DMBA-DM. (a) 4 weeks of exposition (stomatitis). (b) 7 weeks of exposition (fur loss). (c) 10 weeks of exposition (roughness and redness). (d) 13 weeks of exposition (exophytic tumor and papilloma). (e) 15 weeks of exposition (multifocal tumors).

On the contrary, the second experiment with animal immunosuppression had totally different findings. After four weeks of treatment, all the animals from group A (100%) had red thickened mucosa on the exposed pouch. One out of the two animals sacrificed at that time presented severe and extensive ulceration and stomatitis (Figure 2). By exploratory necropsy, the presence of moderate multifocal red lumps was established on the exposed snout of the two sacrificed animals. At the second sacrifice (7 weeks of DMBA/DM exposition), fur loss close to the snout commissure was common in all animals, and three out of the eight (37%) still-alive animals presented moderate to severe multifocal stomatitis and ulceration. By 10 weeks of carcinogenic induction, two out of four (50%) animals presented red papilloma; after 13 weeks, such lesions developed into ulcers and tumors (Figure 2). The exploratory findings after the hamsters' necropsy determined the presence of a red papilloma on the inner side of the pouch of one out of the two animals sacrificed. The last two hamsters to be sacrificed at the end of the 15th week lost around 23% of their body weight in a week. The tumor developed by the last animal to be sacrificed from group A had a volume of 18.7 mm^3^. Furthermore, the last animal euthanized from the control group (no DMBA ) at week 15 lost 24% of its body weight, considering the dexamethasone scheme administered to all the 14 animals (DMBA-exposed and control animals).

**Figure 3:**
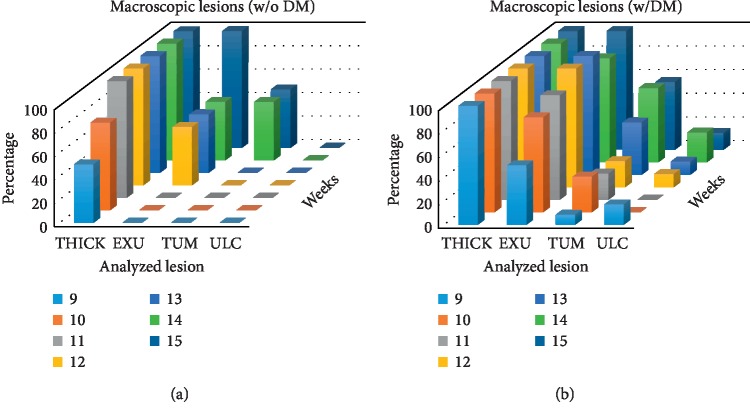
Percentage of animals which presented macroscopic mucosa lesions. Data are shown from week 9 of DMBA exposition. (a) 1st experiment without DM administration. (b) 2nd experiment with DM administration.

Four different lesions: mucosal thickness (THICK), exudative secretion (EXU), tumors (TUM), and ulcers (ULC), were present on the DMBA-exposed hamster cheek pouches. Before 9 weeks of DMBA exposition, there was evidence of the mucosa redness in all exposed animals (data are not relevant to show in figures). Percentage of animals, which presented each of the gross lesions evaluated, is shown in Figure 3; these lesions had relevant incidence during week 9 to week 15.  Figure 3(a) shows gross lesions in animals without corticoid treatment, although it is important to highlight that dexamethasone was administered to the last two animals in the study (weeks 12–15).  Figure 3(b) presents the percentage of animals which presented the lesions evaluated in the second experiment when all the animals were treated with dexamethasone from the beginning.

### 3.2. Histopathological Analysis

No alterations were detected in mucosa tissues from all the negative control hamsters. Analysis of vital organs samples did not show any relevant histopathological lesions.

In the case of the first experiment (no DM administration), DMBA-exposed and control hamsters had the oral mucosa well differentiated with a keratinized thin layer, followed by the epithelium, composed of thin cells and a basal lamina of rounded cells, as it is common to this kind of tissue. After 12 weeks of exposition, there was moderate and multifocal lymphocytic infiltration from connective tissue toward the epithelium associated with animal immune response to carcinogenic DMBA. These inflammatory lesions were inhibiting the morphological changes in the oral epithelium to develop cancer; for this reason, it was decided to use an immunosuppressive agent to prevent the immune response against the inflammatory and the carcinogenic effect of DMBA. After 14 weeks of concomitant DM treatment, only one of these treated animals developed a well-differentiated ulcerated carcinoma with trabeculae and nest of pleomorphic neoplastic squamous cells infiltrating even submucosa. Close to the carcinoma, a papilloma was developed (Figure 1). Histopathological features were analyzed, and frequency of each of the lesions presented on the tissues evaluated after hamsters' necropsies is summarized in Table 1. Each time of evaluation (rows of “weeks of DMBA exposition”) corresponds to two animals, which were euthanized to obtain the tissue samples; in the table, frequencies of lesion appearance are shown. Frequency of lesions developed in the 1st (w/o DM) and in 2nd (w/DM) experiment is listed. 

**Figure 4:**
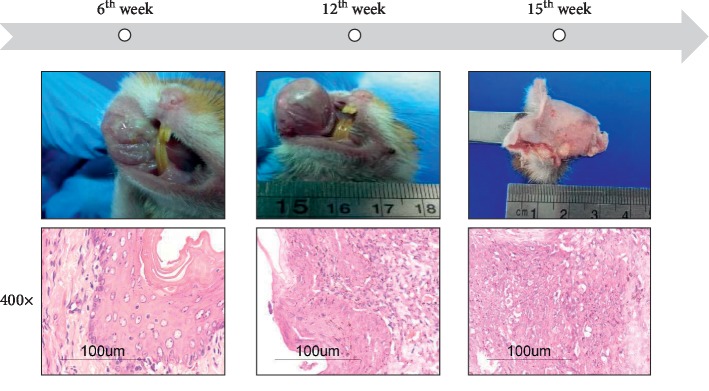
Macroscopic and corresponding microscopic images of OSCC development during DM-DMBA concomitant exposition (H&E stained). At 6, dysplasia and hyperkeratosis were evident. Around 12 weeks, papillomas were found macro- and microscopically. Finally, OSCC and multifocal neoplasias were developed on hamsters' pouch.

In the second experiment, dexamethasone (1 mg/kg) was administered subcutaneously daily for three weeks and this treatment was performed every three weeks; at the same time, DMBA exposition followed the same protocol as described for the first experiment. After 3 weeks of DMBA exposition, there was evidence of hyperkeratinized regions in both of the euthanized hamsters. In addition, there was moderate lymphocytic, histiocytic, and suppurative inflammatory infiltration with mild invasion of mast cells. Evident macroscopic exudation and stomatitis on the mucosa of the exposed pouch were related with histopathological findings of moderate to severe exocytosis, necrosis, and dysplasia of the epithelium (Figure 4). After 6 weeks, moderate to severe exocytosis and necrosis localized in specific regions were found but not on all the mucosa tissue. Mild dysplasia was observed by week 9 in one animal. Although macroscopic evidence shows a tumor in one of the exposed animals at week 12, not all of the four hamsters alive at that moment developed that kind of tumor; microscopic evidence of the euthanized and sampled animals only revealed a mild dysplasia specially developed on the skin of the snout, but no signs of abnormalities were found on mucosa tissue. It is important to note that not all of the exposed animals developed the same lesions and there was an incidence of carcinoma around 50% out of the treated group. The number of animals which developed histopathological lesions at each time of sacrifice is shown in Table 1 (numbers on the right side). At the end of 15 weeks, the last two animals revealed the presence of two different types of tumors developed. The first one was a unique carcinomatous tumor with a volume of 6.24 mm^3^ with the presence of little papilloma (not measured) just under the major neoplasia. The other animal developed a series of multifocal tumors with less volume than that found in the first experiment. In both cases, severe carcinoma *in situ* was developed with the total loss of the architecture, squamous neoplastic cell-infiltrating submucosa, more than 10 mitotic counts (10 fields in 400x), hyperkeratinized regions, and a huge presence of mast cells. Because of immunosuppression, one of these animals got a serious bacterial sepsis evidenced by microscopic analysis. Hemorrhages and neovascularization were also very common on these pathological tissues (Figure 4). Other findings revealed the pathological status; severe ulceration, exocytosis, and necrosis were found during microscopic observation.

The implementation of the successful induction of OSCC was confirmed in a third different assay, exposing 18 animals to DMBA application and subcutaneous dexamethasone treatment. Rapidly, redness and thickness of the mucosa were observed after three weeks of exposition. Tumors macroscopically identified were observed since week 10, and the last animal developed tumor by the 14th week. 15 out of the 18 animals exposed (83%) developed tumors, and volume of these neoplasias is registered in Supplementary Materials (available ([Supplementary-material supplementary-material-1])). Note the volume of tumors is different for each animal, and they are between 2.22 mm^3^ and 119.85 mm^3^. These animals were treated with a synthetic peptide derived from bovine lactoferricin (data to be published).

## 4. Discussion

Several studies have proposed a step-by-step development of the mucosa lesions during administration of DMBA under the same protocol employed here [7]; although there was no macroscopic evidence of tumors by the 12th week in the first experiment conducted, no DMBA-exposed mucosa had a well-differentiated epithelium and basal membrane, with polyhedral cells and a hyperkeratinized outer layer. Carcinogenic-agent exposed pouches, by contrary, presented low hyperplasia and evident signs of lymphocytic infiltration in response to the chronic exposition to the carcinogenic DMBA. These findings were the fact to decide to use an immunosuppressive agent to the last animals exposed; this treatment led to the development of tumors at week 14. In consequence, the second experiment was conducted under the same protocol, using the same DMBA concentration dissolved in mineral oil, using the same technique, and using the same frequency and times of application but with the influence of dexamethasone as described here.

Early studies carried out at the time of standardization of this animal model used, for example, cortisone to induce more invasive tumors and metastatic reaction [17]. The time of initial dysplasia was diminished getting more extensive and larger tumors when 2.0 mg cortisone was administered [16]; even specific anti-lymphocytic serum for hamsters was proved to get an enhanced effect on the carcinoma development [15]. At the same time, other assays, e.g., involving the topical administration of both cortisone acetate (0.05%) and DMBA (0.5%) in a mineral oil solution, revealed the inhibitory effect of the corticoid on SCC development when locally applied [24]. Nowadays, the same contradiction is matter of analysis. Some of the available preclinical models to evaluate the carcinogenic potential of corticoids are not reliable enough; the differences between animal species used, route of administration, and time of corticoid treatment (prior, simultaneous, or after carcinogenic exposition) result in enhancement or inhibition of neoplasia. In general lines, systemic administration of the corticoid at the same time of carcinogenic exposition could result in SCC enhancement [19]. Considering that the precancerous cell microenvironment changes depending on cell transformation to infiltrative carcinogenic cells, immunomodulatory intervention could vary the expression of T cells and interleukins involved in the cancerization process [25]. CD4+ cells infiltrate mainly to the lamina propria of oral leukoplakias; as soon as these lesions transform its infiltrative capacity to turn into carcinoma, mucosa increases the recruitment of CD4+ cells. These cells associated with the transcription regulatory protein Foxp3 (Treg cells) inhibit the antitumor action of IFN-*γ* and MCPs [26]. Glucocorticoids, like dexamethasone, increase the Treg cell expression with a consequent increase of IL-10, an anti-inflammatory cytokine involved in the protection of the cancer cell microenvironment [27]. In our case, dexamethasone administration evidenced an enhanced carcinogenic effect with appearance of tumors around 13 weeks of cancer induction. Another aim of this work was to evaluate the carcinogenic lesions' progress to identify the time of cancer induction necessary to get a carcinoma on oral mucosa; however, this animal model results to be too much variable and time-nonspecific for each step in the carcinogenesis progress. Some animals did not show premalignant lesions (such as leukoplakia, erythroplakia, or proliferative verrucous leukoplakia) prior to neoplasias development. These findings are according to the concept of “field cancerization” proposed by Slaughter and colleagues [28]. Field cancerization refers to that an area of epithelium has been preconditioned by a carcinogenic agent for some period, and it produces irreversible molecular changes in cells with no apparent clinical or histological signs [29]. Oral carcinoma could arise from multifocal areas and not from one cell that suddenly becomes malignant. There were lesions related to dysplasia found at week 4 going directly to well-differentiated carcinoma at the end of week 16.

Additionally, it was possible to implement the animal model of DMBA-induced carcinogenesis in the hamster oral pouch after a failed assay with no visible tumors. Cancerization in this hamster model is comparable to the human process; for example, by 4 weeks of DM-DMBA exposition, necrosis and dysplasia were found microscopically while evidencing macroscopic signs of fibrosis. As seen in the human cancerization, fibrosis is one of the most important precancerous lesions found in patients with several potentials of carcinoma development [30]. Although the use of dexamethasone could implicate some differences with respect to the natural development of cancer, immunosuppression is a suggested alternative when hamsters have the potential to express a strong immune response. In order to obtain an OSCC model to study chemoprevention or potential treatment, dexamethasone immunomodulation could be an interesting enhancer. Other types of studies accidentally found natural tumor enhancers, reporting that carcinoma is developed in 100% of DMBA-exposed animals by 14 weeks when a dose of black coffee was administered via intragastric intubation [31]. Even the carcinoma-promoting effect of Taiwan betel quid was demonstrated increasing the number and size of tumors when hamster pouches were preexposed to DMBA [32]. Another natural cofactor that accelerates the DMBA carcinogenesis is temperature; Kathiresan and Sithrangaboopathy found an earlier incidence of tumors when hamsters were housed at 28°C [33].

Interesting findings include the massive infiltration of mast cells especially when the carcinoma is totally developed. Aromando et al. found that mast cells are highly activated when cell proliferation is increased mediating the release of tryptase, suggesting a new biomolecular target in OSCC [34]. It is expected that further investigations about the role of markers could be included in the development of the present assays.

## Figures and Tables

**Table 1:** Frequency of histopathological lesions observed at each time of sacrifice.

Weeks of DMBA exposure	Number of animals with histopathological lesions													
	LymSubInf		EXO		ULC		NEC		DYS		PAP		SCC	
	w/o DM	w/DM	w/o DM	w/DM	w/o DM	w/DM	w/o DM	w/DM	w/o DM	w/DM	w/o DM	w/DM	w/o DM	w/DM
3	1	2	—	2	—	—	—	—	—	—	—	—	—	—
6	1	1	—	1	—	—	1	1	—	—	—	—	—	—
9	2	1	2	—	—	—	1	1	—	1	—	—	—	—
12	2	—	2	—	—	—	2	1	—	1	—	1	—	—
15	2	1	2	1	1	2	1	2	1	2	1	2	1	2

w/o DM: without dexamethasone; w/DM: with dexamethasone; LymSubInf = lymphocytic submucosal infiltration; EXO = exocytosis; ULC = ulceration; NEC = necrosis; DYS = dysplasia; PAP = papilloma; SCC = squamous cell carcinoma.

## Data Availability

Figures, photographs, and microphotographs used to support the findings of this study are included within the article. Additionally, supplementary materials have been provided.

## Abbreviations

DMBA: 7,12-Dimethylbenzanthracene
DM: Dexamethasone
OSCC: Oral squamous cell carcinoma.

## Supplementary Materials

Supplementary Materials Supplementary Figure 1: submucosal lymphocytic infiltration after 12 weeks of DMBA exposition (no DM was administered, right) compared to normal mucosa of the not-treated hamster buccal pouch (left) (H&E stained). Supplementary Table 1: volume of neoplasia in the mucosa oral cavity with DM-DMBA exposition.
