# Supplementary material for: DMBA-Induced Oral Carcinoma in Syrian Hamster: Increased Carcinogenic Effect by Dexamethasone Coexposition
Source: Biomed Res Int. 2020 Feb 13;2020:1470868. doi: 10.1155/2020/1470868 (PMC7042540; doi:10.1155/2020/1470868)
Supplement: Supplementary Materials — Supplementary Figure 1: submucosal lymphocytic infiltration after 12 weeks of DMBA exposition (no DM was administered, right) compared to normal mucosa of the not-treated hamster buccal pouch (left) (H&E stained). Supplementary Table 1: volume of neoplasia in the mucosa oral cavity with DM-DMBA exposition. [file 1470868.f1.docx]

**Supplementary material**

| Normal mucosa | Lymphocytic infiltration |
| --- | --- |
| 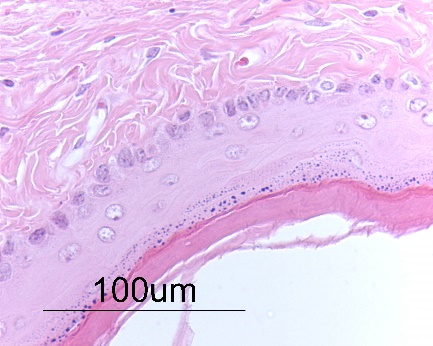 | 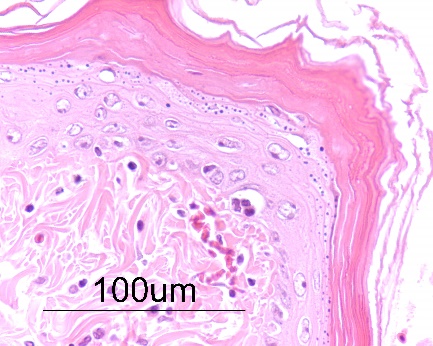 |

**Supplementary Figure 1.** Submucosal lymphocytic infiltration after 12 weeks of DMBA exposure (no DM administrated, right) compared to normal mucosa of no treated hamster buccal pouch (left). H&E stained.

**Supplementary Table 1**

Volume of neoplasia in mucosa oral cavity with DM-DMBA exposure.

| Animal # | Tumor Vol  (mm^3^) | Animal # | Tumor Vol  (mm^3^) | Animal # | Tumor Vol  (mm^3^) |
| --- | --- | --- | --- | --- | --- |
| 1 | 17.05 | **6** | 29.17 | **11** | 12.08 |
| 2 | 5.55 | **7** | 3.79 | **12** | 2.22 |
| 3 | 6.71 | **8** | 5.67 | **13** | 6.25 |
| 4 | 8.98 | **9** | 6.68 | **14** | 3.08 |
| 5 | 30.71 | **10** | 4.85 | **15** | 119.85 |

Macroscopic tumors after 10-14 weeks of DMBA exposure.
